# Sex-Specific Risk Factors Associated With First Acute Myocardial Infarction in Young Adults

**DOI:** 10.1001/jamanetworkopen.2022.9953

**Published:** 2022-05-03

**Authors:** Yuan Lu, Shu-Xia Li, Yuntian Liu, Fatima Rodriguez, Karol E. Watson, Rachel P. Dreyer, Rohan Khera, Karthik Murugiah, Gail D’Onofrio, Erica S. Spatz, Khurram Nasir, Frederick A. Masoudi, Harlan M. Krumholz

**Affiliations:** 1 Center for Outcomes Research and Evaluation, Yale New Haven Hospital, New Haven, Connecticut; 2 Section of Cardiovascular Medicine, Department of Internal Medicine, Yale School of Medicine, New Haven, Connecticut; 3 Division of Cardiovascular Medicine, School of Medicine, Stanford University, Stanford, California; 4 David Geffen School of Medicine, University of California, Los Angeles; 5 Department of Emergency Medicine, Yale School of Medicine, New Haven, Connecticut; 6 Department of Biostatistics, Health Informatics, Yale School of Public Health, New Haven, Connecticut; 7 Division of Cardiology, UT Southwestern Medical Center, Dallas, Texas; 8 Division of Cardiovascular Prevention and Wellness, Houston Methodist DeBakey Heart and Vascular Center, Houston, Texas; 9 Center for Outcomes Research, Houston Methodist Research Institute, Houston, Texas; 10 Research and Analytics, Ascension Health, St Louis, Missouri; 11 Department of Health Policy and Management, Yale School of Public Health, New Haven, Connecticut

## Abstract

**Importance:**

An increasing proportion of people in the US hospitalized for acute myocardial infarction (AMI) are younger than 55 years, with the largest increase in young women. Effective prevention requires an understanding of risk factors associated with risk of AMI in young women compared with men.

**Objectives:**

To assess the sex-specific associations of demographic, clinical, and psychosocial risk factors with first AMI among adults younger than 55 years, overall, and by AMI subtype.

**Design, Setting, and Participants:**

This study used a case-control design with 2264 patients with AMI, aged 18 to 55 years, from the VIRGO (Variation in Recovery: Role of Gender on Outcomes of Young AMI Patients) study and 2264 population-based controls matched for age, sex, and race and ethnicity from the National Health and Nutrition Examination Survey from 2008 to 2012. Data were analyzed from April 2020 to November 2021.

**Exposures:**

A wide range of demographic, clinical, and psychosocial risk factors.

**Main Outcomes and Measures:**

Odds ratios (ORs) and population attributable fractions (PAF) for first AMI associated with demographic, clinical, and psychosocial risk factors.

**Results:**

Of the 4528 case patients and matched controls, 3122 (68.9%) were women, and the median (IQR) age was 48 (44-52) years. Seven risk factors (diabetes [OR, 3.59 (95% CI, 2.72-4.74) in women vs 1.76 (1.19-2.60) in men], depression [OR, 3.09 (95% CI, 2.37-4.04) in women vs 1.77 (1.15-2.73) in men], hypertension [OR, 2.87 (95% CI, 2.31-3.57) in women vs 2.19 (1.65-2.90) in men], current smoking [OR, 3.28 (95% CI, 2.65-4.07) in women vs 3.28 (2.65-4.07) in men], family history of premature myocardial infarction [OR, 1.48 (95% CI, 1.17-1.88) in women vs 2.42 (1.71-3.41) in men], low household income [OR, 1.79 (95% CI, 1.28-2.50) in women vs 1.35 (0.82-2.23) in men], hypercholesterolemia [OR, 1.02 (95% CI, 0.81-1.29) in women vs 2.16 (1.49-3.15) in men]) collectively accounted for the majority of the total risk of AMI in women (83.9%) and men (85.1%). There were significant sex differences in risk factor associations: hypertension, depression, diabetes, current smoking, and family history of diabetes had stronger associations with AMI in young women, whereas hypercholesterolemia had a stronger association in young men. Risk factor profiles varied by AMI subtype, and traditional cardiovascular risk factors had higher prevalence and stronger ORs for type 1 AMI compared with other AMI subtypes.

**Conclusions and Relevance:**

In this case-control study, 7 risk factors, many potentially modifiable, accounted for 85% of the risk of first AMI in young women and men. Significant differences in risk factor profiles and risk factor associations existed by sex and by AMI subtype. These findings suggest the need for sex-specific strategies in risk factor modification and prevention of AMI in young adults. Further research is needed to improve risk assessment of AMI subtypes.

## Key Points

**Question:**

What are the sex-specific associations of demographic, clinical, and psychosocial risk factors with first acute myocardial infarction (AMI) among adults younger than 55 years, overall, and by AMI subtype?

**Findings:**

In this matched case-control study that included 2264 patients with AMI (aged 18-55 years) and 2264 population-based controls, 7 risk factors, many potentially modifiable, accounted for 85% of the risk of first AMI in young women and men. Significant differences in risk factor profiles and risk factor associations existed by sex and by AMI subtype.

**Meaning:**

Significant sex differences in risk factor profiles suggest the need for sex-specific strategies in risk factor modification and prevention of AMI in young adults.

## Teaser

This case-control study assesses the sex-specific associations of demographic, clinical, and psychosocial risk factors with first acute myocardial infarction among adults younger than 55 years, overall, and by infarction subtype.

## Introduction

Approximately 800 000 people in the US are hospitalized for acute myocardial infarction (AMI) each year, of whom a third are younger than 55 years.^1^ The proportion of AMI hospitalizations attributable to younger individuals has been increasing steadily, making heart disease a leading cause of mortality and morbidity in this age group.^2,3^ However, the risk factors for development of AMI and their relative importance by sex and subtypes of AMI in younger ages are not well characterized, limiting the effectiveness of efforts to identify and treat at-risk individuals.

Although previous studies and reviews have assessed risk factors for AMI in younger individuals, they have several limitations.^4,5,6,7,8,9,10,11,12,13,14,15,16,17,18,19^ Most studies have exclusively included people with AMI without a control population. Therefore, they could only evaluate the prevalence of risk factors in people with AMI and were limited in making inferences about risk factors and their relative importance in young women and men.^4,5,6,7,8,9,10,11^ The few studies that assessed the strength of association between risk factors and AMI^12,13,14,15,16,17,18,19^ included only men, or only women, or older adults; ascertained AMI cases by self-report or diagnosis coding without rigorous adjudication; evaluated only a couple of risk factors at a time; or were unable to classify AMI subtypes.^20,21^ Developing a deeper understanding of a wide range of risk factors associated with AMI in young women and men and the strength of these associations, as well as identifying how these associations vary by sex and AMI subtype, has important implications for designing primary prevention strategies targeted to young adults.

Accordingly, we used a case-control design with 2264 patients with AMI from the VIRGO (Variation in Recovery: Role of Gender on Outcomes of Young AMI Patients) study^22^ and 2264 population-based controls matched for age, sex, and race and ethnicity from the National Health and Nutrition Examination Survey (NHANES) population to examine the associations of a wide range of risk factors on first AMI in young adults and to ascertain if the associations vary by sex and AMI subtype. To the best of our knowledge, this is the largest study in the US that focused on young women and a comparable sample of similarly aged men and comprehensively evaluated the associations between a wide range of predisposing risk factors and incident AMI by sex. The novelty of this study is the design of using NHANES as a control population and then, with matching, the determination of what factors are most important for this population. We hypothesized that there were significant differences in risk factor profiles and risk factor associations by sex.

## Methods

### Study Population

The study population included patients with AMI from the VIRGO study^22^ and population-based controls matched for age, sex, and race and ethnicity from the NHANES population. Because AMI had a long latent period for disease manifestation and the incidence rate of AMI was low in young individuals, we chose a case-control study design to test multiple potential risk factors for AMI.^23^ This study was approved by the Institutional Review Board at Yale University and patients provided written informed consent for their study participation. The study followed the guidelines for cohort studies, described in Strengthening the Reporting of Observational Studies in Epidemiology (STROBE) reporting guidelines for case-control studies.

The VIRGO study is the largest prospective observational study of young women and men with AMI in the US.^22^ We recruited all women aged 18 to 55 years presenting with an AMI to 103 geographically diverse hospitals across the US between August 2008 and January 2012. We recruited a random sample of men with AMI from these hospitals to achieve a 2:1 female-to-male enrollment ratio according to the prespecified protocol. A total of 2985 patients were finally enrolled. The characteristics of VIRGO participants were similar to those of patients with AMI in the US National Inpatient Sample.^4^ Consistent with the third Universal Definition of MI,^24,25^ we defined AMI as an increased cardiac biomarker level indicative of myocardial necrosis (at least 1 cardiac biomarker >99th percentile of the upper reference limit) within 24 hours of admission and symptoms of ischemia, electrocardiogram changes indicative of new ischemia, or imaging evidence of infarction. Independent adjudication of AMI subtypes was performed by a team of 5 physicians as previously described.^26^ The classification of AMI subtypes was based on physicians’ explicit review of several clinical domains: (1) risk factors and clinical presentation, (2) electrocardiography, (3) angiographic findings, (4) left ventricular systolic function, and (5) magnitude of troponin elevation, as well as the reviewers’ impression of the primary and secondary pathophysiological mechanisms underlying the AMI. We defined type 1 AMI as caused by plaque rupture, ulceration, fissuring, erosion, or dissection with resulting thrombus.^25^ Type 2, type 4b, and unclassified AMI were combined into the “other” category. Type 3, type 4a, and type 5 AMI were excluded from the VIRGO study (eTable 1 in the Supplement). Because we focused on patients with the first AMI, we excluded patients with previous AMI (n = 476).

Population-based controls were derived from NHANES, a series of cross-sectional surveys that provide a nationally representative sample of the noninstitutionalized population in the US.^27^ We excluded people with a self-reported history of cardiovascular diseases, defined as individuals with a history of coronary heart disease, angina or angina pectoris, MI, or stroke. Controls and cases were matched 1:1 on the basis of age, sex, and race and ethnicity in the main analysis. We included participants from 3 rounds of NHANES conducted between 2007 and 2012 to obtain temporally concurrent controls.

### Data Collection

In VIRGO,^22^ information on patients’ sociodemographic characteristics, lifestyle, family history of cardiovascular diseases, and psychosocial factors was collected by standardized in-person interviews administered by trained personnel during the index AMI admission. Information on medical history, medication use on admission, physical measurements, lipid profile, and hemoglobin A_1c_ level were collected from medical record abstraction. In NHANES, in-person interviews were conducted by trained interviewers to collect participants’ information on sociodemographic characteristics, medical history, family history of cardiovascular diseases, and psychosocial factors. Physical examination, including physical measurements and laboratory tests, was also administered by trained medical personnel in a random subsample of participants. In both VIRGO and NHANES, self-reported physical activity was measured using the Behavioral Risk Factor Surveillance Survey physical activity instrument.^28^ Symptoms of depression that patients experienced in the past 2 weeks were assessed during the index AMI admission using the 9-item version of the Patient Health Questionnaire (PHQ-9).^29^

### Variable Definitions

We used consistent definitions of risk factors in VIRGO and NHANES (eTable 2 in the Supplement). In both studies, history of hypertension was defined by previous physician diagnosis or currently on antihypertensive medication. We did not use blood pressure levels because among those with AMI, blood pressure could be influenced by the characteristics of the AMI or by evidence-based therapies. Consistent with the American Diabetes Association guideline,^30^ history of diabetes was defined by previous physician diagnosis, or hemoglobin A_1c_ level 6.5% or greater, or currently receiving antidiabetic medication. History of hypercholesterolemia was defined by previous physician diagnosis, or total cholesterol level 200 mg/dL or greater, or low-density lipoprotein cholesterol level 130 mg/dL or greater, or high-density lipoprotein cholesterol less than 40 mg/dL, or currently receiving lipid-lowering medication (to convert cholesterol to millimoles per liter, multiply by 0.0259). Obesity was defined as body mass index, calculated as weight in kilograms divided by height in meters squared, 30 or greater. High waist circumference was defined as waist circumference greater than 88 cm for women and greater than 102 cm for men. Smoking status was classified into current smokers, former smokers, and never smokers. Physical activity was classified into 2 levels: recommended physical activity (150 minutes or more of moderate physical activity or 75 minutes or more of vigorous physical activity per week) or insufficient physical activity or inactivity (less than 149 minutes per week of moderate physical activity or less than 74 minutes per week of vigorous physical activity).^31^ Consistent with previous studies, regular alcohol intake was defined as consumption 3 or more times a week.^12^ Family history of premature MI was defined as having any blood relatives who had an MI before the age of 50 years. Presence of depressive symptoms was defined as PHQ-9 score 10 or greater. Early menopause was defined as a woman reaching menopause before age 45 years.

### Statistical Analysis

We used multivariable conditional logistic regression to quantify the association of various risk factors with AMI and adjusted for all covariates that were significant in the univariable analysis. We did not include age, sex, and race and ethnicity as covariates in the models because these variables were adjusted for by matching. Given the focus of sex-specific risk factors for AMI, we developed separate models for men and women and compared the prevalence and odds ratio (OR) of risk factors for men vs women. To test the interaction between sex and risk factors, we developed unconditional logistic regressions with interaction terms between sex and risk factors as well as interaction terms between matching factors because conditional logistic regressions did not allow matching factors to interact with explicitly modeled variables.^32^ We performed the sex-specific analyses for overall AMI outcome and then further stratified by type 1 AMI vs other AMI subtypes.

We estimated the combined effect of multiple risk factors by deriving the adjusted ORs for combination of risk factors through summation of their respective model coefficients and exponentiation in the multivariable logistic model. We calculated population attributable fraction (PAF), an estimation of the fraction of AMI cases in the population that were attributable to an exposure to 1 or several risk factors, using appropriate methods for case-control studies (see eMethods in the Supplement).^33^

Missing data were assumed to be missing at random. For covariates considered in each model, missing values were rare (<5% in the study cohort). Missing values were imputed as the most common values for categorical variables and as median values for continuous variables.^34^ Statistical significance was defined as a *P* < .05 for 2-sided tests. Analyses were conducted in R, version 4.0 (R Foundation for Statistical Computing).

## Results

A total of 2264 patients with a first AMI in VIRGO and 2264 age-, sex-, and race and ethnicity–matched controls in NHANES were included, of whom the median (IQR) age was 48 (44-52) years, and 3122 (68.9%) were women; 210 (4.6%) were Hispanic individuals, 734 (16.2%) were non-Hispanic Black individuals, 3410 (75.3%) were non-Hispanic White individuals, and 174 (3.8%) were of other race or ethnicity (including Asian individuals, Native American individuals, and individuals of other races). Among patients with AMI, 1861 (82.2%) had type 1 AMI, 86 (3.8%) had type 2 AMI, and 317 (14.0%) had other subtypes; 76 (3.4%) had a history of cancer (Table 1).

**Table 1.** Characteristics of Study Population by Sex

Characteristics	No. (%)^a^						*P* value for difference between men and women among cases
	Men			Women			
	Cases (n = 703)	Controls (n = 703)	*P* value for difference between cases and controls	Cases (n = 1561)	Controls (n = 1561)	*P* value for difference between cases and controls	
Sociodemographic characteristics							
Age, median (IQR), y	48 (43-52)	48 (43-52)	>.99	48 (44-52)	48 (44-53)	.90	.53
Race and ethnicity							
Hispanic	34 (4.8)	34 (4.8)	>.99	71 (4.5)	71 (4.5)	>.99	<.001
Non-Hispanic							
Black	73 (10.4)	73 (10.4)		294 (18.8)	294 (18.8)		
White	572 (81.4)	572 (81.4)		1133 (72.6)	1133 (72.6)		
Other^b^	24 (3.4)	24 (3.4)		63 (4.0)	63 (4.0)		
Married/living with a partner as if married	442 (62.9)	469 (66.7)	.15	836 (53.6)	973 (62.4)	<.001	<.001
Education							
Less than high school	3 (0.4)	122 (17.4)	<.001	20 (1.3)	255 (16.4)	<.001	.17
High school	282 (40.5)	175 (24.9)		627 (40.4)	347 (22.3)		
More than high school	411 (59.1)	406 (57.8)		904 (58.3)	957 (61.4)		
Annual household income, $							
<10 000	86 (13.1)	40 (65.8)	<.001	304 (20.5)	103 (6.9)	<.001	<.001
10 000-99 000	435 (66.0)	468 (68.2)		1031 (69.4)	1063 (70.9)		
≥100 000	138 (20.9)	178 (25.9)		151 (10.2)	333 (22.2)		
Health insurance	536 (76.6)	528 (75.1)	.56	1236 (79.3)	12 474 (79.7)	.84	.16
Insurance covers prescription	502 (72.3)	504 (71.9)	.90	1171 (75.5)	1192 (76.6)	.52	.12
Comorbidities and CVD risk factors							
Hypertension	412 (58.6)	239 (34.0)	<.001	978 (62.7)	444 (28.5)	<.001	.075
Diabetes	161 (22.9)	84 (12.6)	<.001	580 (37.2)	151 (10.3)	<.001	<.001
Hypercholesterolemia	642 (91.3)	517 (76.9)	<.001	1275 (81.7)	1033 (70.2)	<.001	<.001
Obesity (BMI ≥30)	342 (48.6)	237 (35.1)	<.001	839 (53.7)	610 (40.6)	<.001	.03
High waist circumference (women, >88 cm; men, >102 cm)	297 (52.1)	310 (47.0)	.08	988 (81.1)	1008 (69.6)	<.001	<.001
Smoking status							
Never	212 (30.2)	352 (50.2)	<.001	453 (29.0)	871 (55.8)	<.001	.21
Former	123 (17.5)	158 (22.5)		236 (15.1)	311 (19.9)		
Current	367 (52.3)	191 (27.2)		872 (55.9)	378 (24.2)		
Live with anyone who smokes	248 (35.6)	142 (20.3)	<.001	626 (40.3)	297 (19.2)	<.001	.04
Regular alcohol intake	151 (21.9)	161 (22.9)	.72	161 (10.6)	181 (11.6)	.39	<.001
Physical activity							
Recommended	324 (46.6)	507 (72.1)	<.001	537 (34.7)	913 (58.5)	<.001	<.001
Insufficient	181 (26.0)	87 (12.4)		470 (30.4)	268 (17.2)		
Inactive	191 (27.4)	109 (15.5)		542 (34.9)	380 (24.3)		
History of congestive heart failure	9 (1.3)	5 (0.7)	.42	46 (2.9)	12 (0.8)	<.001	.025
Family history of premature MI	202 (30.3)	77 (11.3)	<.001	459 (30.9)	222 (14.5)	<.001	.842
Family history of diabetes	295 (42.8)	281 (40.7)	.45	819 (53.2)	650 (42.2)	<.001	<.001
Depression	140 (20.6)	51 (8.3)	<.001	588 (39.1)	165 (12.4)	<.001	<.001
Menopause before age 45 y	0	0	NA	91 (6.1)	48 (3.6)	.003	NA
Medication use							
Use of statin	129 (18.3)	92 (13.1)	.008	318 (20.4)	177 (11.3)	<.001	.29
Use of β-blocker	96 (13.7)	54 (7.7)	<.001	265 (17.0)	107 (6.9)	<.001	.05
Use of aspirin	119 (16.9)	5 (0.7)	<.001	288 (18.4)	8 (0.5)	<.001	.42

Abbreviations: BMI, body mass index, calculated as weight in kilograms divided by height in meters squared; CVD, cardiovascular disease; MI, myocardial infarction.

^a^ All percentages were calculated by excluding missing, do not know, and patient refused.

^b^ Other races include Asian individuals, Native American individuals, and individuals of other races.

### Sex-Specific Associations of Risk Factors With AMI in Young Adults

**Figure 1.**
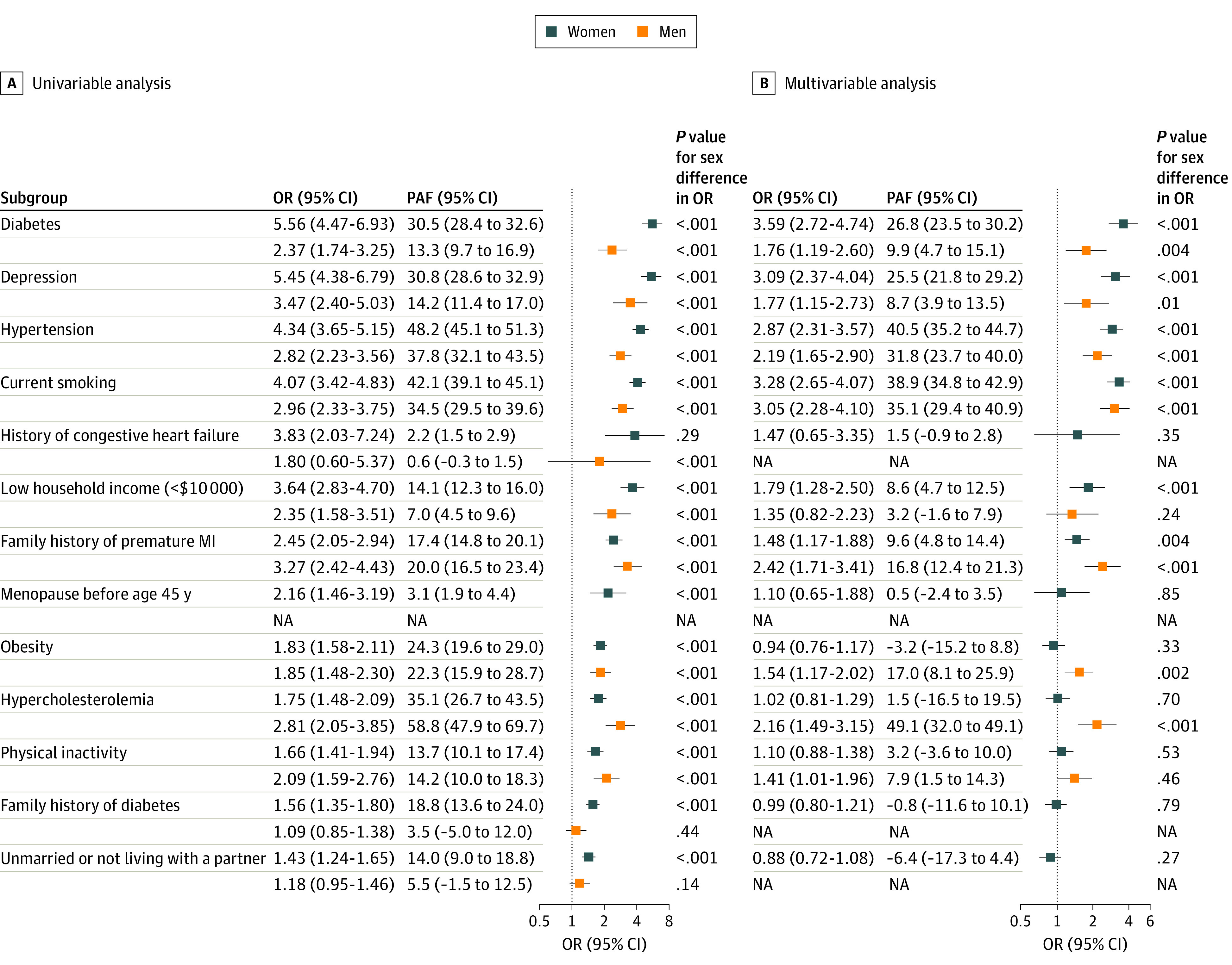
Association of Risk Factors With AMI in Women vs Men Only risk factors that were significant in the univariable analysis were included in the multivariable analysis. Age and race and ethnicity were adjusted by matching. AMI indicates acute myocardial infarction; OR, odds ratio; PAF, population attributable fraction.

In the univariable analyses, 13 risk factors were significantly associated with higher odds of AMI among women, while 9 risk factors were significantly associated with higher odds of AMI among men (Figure 1A). Significant sex interactions were observed for diabetes, depression, hypertension, current smoking, family history of diabetes, and hypercholesterolemia, and these risk factors had stronger associations with AMI in women compared with men, except for hypercholesterolemia, which had stronger associations in men (Figure 1A).

After multivariable adjustment, 7 risk factors (diabetes, depression, hypertension, current smoking, family history of premature MI, low household income, hypercholesterolemia) remained statistically significant in men or women. Specifically, diabetes was associated with the highest odds of AMI among women, followed by current smoking, depression, hypertension, low household income, and family history of premature MI (Figure 1B). Current smoking was associated with the highest odds of AMI among men, followed by family history of premature MI, hypertension, hypercholesterolemia, depression, diabetes, obesity, and physical inactivity. Low household income was significantly associated with AMI in women but not in men, whereas hypercholesterolemia and physical inactivity were significantly associated with AMI in men but not in women.

The contribution of individual risk factors to the total risk of AMI varied between men and women. The PAFs of diabetes (26.8% vs 9.9%), depression (25.5 vs 8.7%), hypertension (40.5% vs 31.8%), and current smoking (38.9% vs 35.1%) were greater among women compared with men, whereas the PAFs of hypercholesterolemia (49.1% vs 1.5%) and family history of premature MI (16.8% vs 9.6%) were greater among men compared with women. Four preventable risk factors (current smoking, hypertension, diabetes, and depression) had a combined PAF of 80.2% in women compared with 63.2% in men. All 7 risk factors (diabetes, depression, hypertension, current smoking, family history of premature MI, low household income, hypercholesterolemia) had a PAF of 83.9% in women and a PAF of 85.1% in men.

### Difference in Risk by AMI Subtype

**Figure 2.**
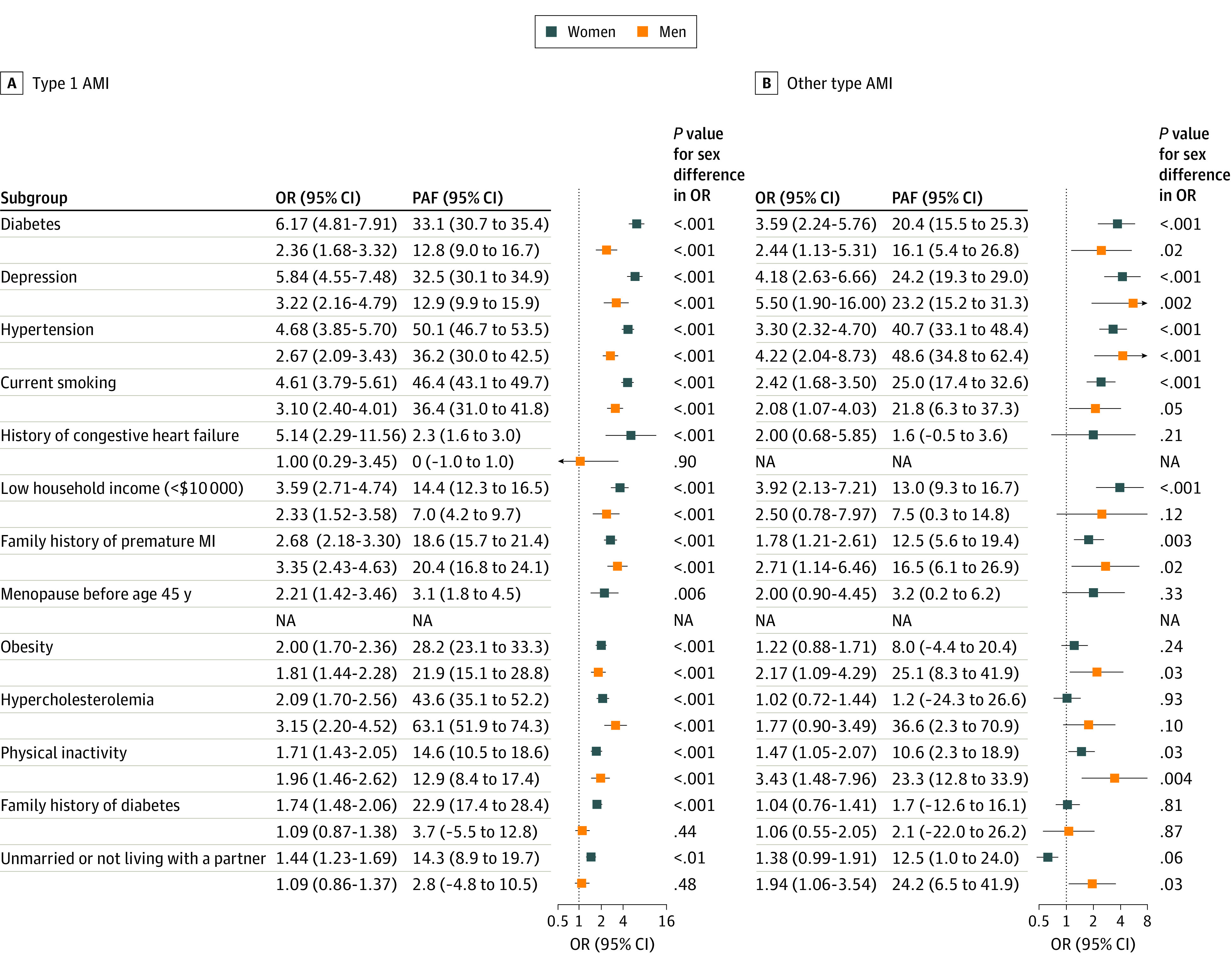
Association of Risk Factors With Acute Myocardial Infarction (AMI) in Type 1 AMI vs Other Types of AMI, Adjusted for Age, Sex, and Race and Ethnicity Age, sex, and race were adjusted by matching. OR indicates odds ratio; PAF, population attributable fraction.

The distribution of risk factors varied significantly by AMI subtype (Table 2). Compared with type 1 AMI, other types of AMI were overrepresented in young women (78.2% vs 67.0%; *P* < .001) and Black individuals (19.1% vs 15.6%; *P* = .007). Traditional cardiovascular risk factors, such as diabetes (33.7% vs 28.0%), hypercholesteremia (86.6% vs 75.7%), obesity (53.8% vs 44.7%), and current smoking (57.4% vs 42.4%), had significantly higher prevalence in patients with type 1 AMI than in those with other types of AMI (*P* < .05 for all; Figure 2). These risk factors were also more strongly associated with type 1 AMI for both men and women (Figure 2).

**Table 2.** Characteristics of Study Population Stratified by Type of AMI

Characteristics	No. (%)^a^				*P* value for difference between AMI subtypes among cases
	Type 1 AMI		Other types of AMI^b^		
	Cases (n = 1861)	Controls (n = 1861)	Cases (n = 403)	Controls (n = 403)	
Sociodemographic characteristics					
Age, median (IQR), y	48 (44-52)	48 (44-52)	48 (42-52)	48 (42-52)	.13
Women	1246 (67.0)	1246 (67.0)	315 (78.2)	315 (78.2)	<.001
Race and ethnicity					
Hispanic	80 (4.3)	80 (4.3)	25 (6.2)	25 (6.2)	.007
Non-Hispanic					
Black	290 (15.6)	290 (15.6)	77 (19.1)	77 (19.1)	
White	1427 (76.7)	1427 (76.7)	278 (69.0)	278 (69.0)	
Other^c^	64 (3.4)	64 (3.4)	23 (5.7)	23 (5.7)	
Married/living with a partner as if married	1061 (57.1)	1187 (63.9)	215 (53.3)	264 (65.5)	.18
Education					
Less than high school	15 (0.8)	330 (17.8)	8 (2.0)	80 (19.9)	.09
High school	745 (40.3)	429 (23.1)	164 (41.1)	84 (20.8)	
More than high school	1088 (58.9)	1100 (59.2)	227 (56.9)	239 (59.3)	
Annual household income, $					
<10 000	324 (18.3)	117 (6.5)	66 (17.5)	21 (5.4)	.34
10 000-99 000	1197 (67.7)	1248 (69.4)	269 (71.2)	277 (70.7)	
≥100 000	246 (13.9)	432 (24.0)	43 (11.4)	94 (24.0)	
Health insurance	1439 (77.6)	1464 (78.7)	333 (82.6)	309 (76.7)	.03
Insurance covers prescription	1366 (74.0)	1411 (76.1)	307 (77.3)	296 (73.6)	.18
Comorbidities and CVD risk factors					
Hypertension	1150 (61.8)	541 (29.1)	240 (59.6)	115 (28.5)	.43
Diabetes	628 (33.7)	199 (11.5)	113 (28.0)	34 (9.1)	.03
Hypercholesterolemia	1612 (86.6)	1289 (74.1)	305 (75.7)	256 (68.6)	<.001
Obesity (BMI ≥30)	1001 (53.8)	666 (37.2)	180 (44.7)	140 (36.4)	.001
High waist circumference (women, >88 cm; men, >102 cm)	1064 (72.1)	1054 (60.9)	221 (70.8)	238 (64.3)	.71
Smoking status					
Never smoker	504 (27.1)	966 (52.0)	161 (40.0)	223 (55.3)	<.001
Former smoker	288 (15.5)	398 (21.4)	71 (17.6)	90 (22.3)	
Current smoker	1068 (57.4)	495 (26.6)	171 (42.4)	90 (22.3)	
Live with anyone who smokes	746 (40.3)	380 (20.5)	128 (32.0)	71 (17.7)	.002
Regular alcohol intake	259 (14.2)	308 (16.6)	53 (13.5)	55 (13.6)	.77
Physical activity					
Recommended	686 (37.2)	1158 (44.3)	175 (43.8)	247 (61.3)	<.001
Insufficient	559 (30.3)	296 (15.9)	92 (23.0)	62 (15.4)	
Inactive	598 (32.4)	407 (21.9)	133 (33.2)	94 (23.3)	
History of congestive heart failure	41 (2.2)	17 (0.9)	14 (3.5)	1 (0.2)	.19
Family history of premature MI	548 (31.0)	245 (13.4)	113 (29.4)	71 (17.9)	.59
Family history of diabetes	931 (50.9)	758 (41.3)	183 (45.8)	165 (41.7)	.07
Depression	603 (33.7)	191 (11.7)	125 (32.0)	36 (10.4)	.56
Menopause before age 45 y	71 (6.0)	41 (3.8)	20 (6.6)	9 (3.4)	.85
Medication use					
Use of statin	361 (19.4)	203 (10.9)	86 (21.3)	38 (9.4)	.41
Use of β-blocker	292 (15.7)	136 (7.3)	69 (17.1)	31 (7.7)	.52
Use of aspirin	339 (18.2)	12 (0.6)	68 (16.9)	3 (0.7)	.57

Abbreviations: AMI, acute myocardial infarction; BMI, body mass index, calculated as weight in kilograms divided by height in meters squared; CAD, coronary artery disease; CVD, cardiovascular disease; MI, myocardial infarction.

^a^ All percentages are calculated by excluding missing, do not know, and patient refused.

^b^ Other types of AMI include type 2 (condition other than CAD contributes to imbalance between myocardial oxygen supply or demand), type 4b (stent thrombosis), and unclassified.

^c^ Other races include Asian individuals, Native American individuals, and individuals of other races.

There were sex differences in risk factor associations and PAFs by AMI subtype (Figure 2). Diabetes (OR: 6.17 vs 2.36; PAF: 33.1% vs 12.8%), depression (OR: 5.84 vs 3.22; PAF: 32.5% vs 12.9%), hypertension (OR: 4.68 vs 2.67; PAF: 50.1% vs 36.2%), current smoking (OR: 4.61 vs 3.10; PAF: 46.4% vs 36.4%), family history of diabetes (OR: 1.74 vs 1.09; PAF: 22.9% vs 3.7%), and history of congestive heart failure (OR: 5.14 vs 1.0; PAF: 2.3% vs 0%) were more strongly associated with type 1 AMI in women compared with men, whereas hypercholesterolemia (OR: 2.09 vs 3.15; PAF: 43.6% vs 63.1%) was more strongly associated with type 1 AMI in men. The risk factor associations with other types of AMI were generally similar in men and women except for physical inactivity, which was more strongly associated with men than women (OR: 1.47 vs 3.43; PAF: 10.6% vs 23.3%).

## Discussion

In this case-control study, 7 risk factors (diabetes, depression, hypertension, current smoking, family history of premature MI, low household income, hypercholesterolemia) accounted for 85% of the risk of first AMI in young men and women. We further found that risk factors accounted for different risk of AMI in women compared with men, and that some of the significant factors varied by sex. Risk factors also varied by subtype of AMI, with traditional cardiovascular risk factors having higher prevalence and stronger associations for type 1 AMI compared with other types of AMI not resulting from acute plaque rupture.

This study extends the field in 3 important ways. First, compared with studies conducted in younger individuals, such as the Coronary Artery Risk Development in Young Adults (CARDIA) study and the Framingham Offspring Study,^35,36,37,38^ we included a much larger number of patients with AMI and examined the associations of a wide range of metabolic, familial, and social risk factors that had previously not been fully assessed, providing a more comprehensive picture of risk factors for AMI in young people. We showed that family history of premature MI has a strong association with risk of AMI in young adults, even after adjusting for other covariates. This finding is in contrast to prior observations in older populations in which the association of family history is negligible after accounting for prevalent risk factors,^12^ highlighting the importance of inheritable risk for AMI among younger adults.^39^ The associations of depression and low household income are also strong after adjusting for other risk factors, suggesting that both socioeconomic and psychological factors play an important role in the development of AMI in young individuals, particularly young women.^21^

Second, this study identifies significant sex differences in risk factors associated with AMI and in the strength of associations among young adults. While sex differences in the presence of risk factors among patients with AMI have been extensively studied,^2,9,10^ there is less evidence for the relative importance of pre-event risk factors in this young AMI population. The INTERHEART study, which focused on older populations, observed that hypertension and diabetes had stronger associations in women compared with men, and significant sex interactions were noted.^12,13^ We provide an independent validation of these observations in young adults and further show that current smoking and gender-related characteristics, such as depression and low income,^40^ were associated with a greater OR and PAF in women compared with men. Given that the prevalence of these modifiable risk factors is increasing in the US,^4^ the high prevalence of risk factors and the strong association with AMI in young women together will put them at a substantial risk for future cardiovascular events.

Third, based on rigorous adjudication of AMI cases, our analysis by AMI subtype provides new insights into the epidemiology of etiologically distinct classes of myocardial injury in young adults. Despite the increasing recognition of varying types of AMI in recent years, evidence on the epidemiology, including patient characterization and associated risk factors, is lacking.^41^ We demonstrate significant differences in risk factor profiles and their associations with outcome between subtypes of AMI. We also show that type 2 AMI—a subtype associated with higher mortality^42^—is overrepresented in young women and Black individuals. Moreover, given the breadth of clinical phenotypes in young women, we previously developed a new taxonomy for classifying diverse phenotypic presentations of young women with AMI. We found that AMI without obstructive coronary artery stenoses (ie, maximal stenosis of any 1 major epicardial vessel <50%) disproportionally affected young women (11.3% of women vs 2.7% of men).^26^

This study has clinical and public health implications. As family history of MI is a strong risk factor in young individuals, more research is needed to understand the role of familial factors in developing AMI in young adults. Some familial risk factors may portend higher risk, as recent studies have shown high polygenic score to be associated with an increased risk of AMI in this young population.^39^ In addition, this study identifies the need for sex-specific strategies in risk factor modification and prevention of AMI in young women. Beyond traditional risk factors, gender-related characteristics, such as psychological stressors, depression, and poverty, also play a sizeable role in women’s AMI risk, and hence a multifaceted approach targeted to these risk factors is important to improve sex- and gender-based differences in patient outcomes. The implementation of evidence-based guidelines on preventing AMI in women should be expanded to assist clinicians in decision-making, and effective strategies need to be identified to improve optimal delivery of guidelines.^43^ National initiatives, such as the American Heart Association Go Red for Women campaign, should also be expanded to increase awareness about cardiovascular disease risk in young women.^44^ Finally, our results of different risk factors associated with distinct AMI subtypes call for a more precise assessment of AMI risk in young adults. While current risk prediction tools target an aggregate diagnosis of AMI,^45,46^ development of more specific individual patient risk prediction for each AMI subtype will allow for effective application of preventive therapies in an individual patient. Documentation of detailed MI characterization as specified by international consensus^24^ is also critical to facilitate the understanding of the epidemiology, pathophysiology, and subsequent prognostic implications of different AMI subtypes in young adults.

### Limitations

This study has several limitations. First, while VIRGO and NHANES samples were well matched by age, sex, and race and ethnicity, they may have differed in underlying factors that are known to be associated with AMI. The patients with AMI in VIRGO came from 103 geographically diverse hospitals across the US, and their characteristics were similar to those of patients with AMI nationally.^4^ Therefore, the source population that gave rise to the patients with AMI included in the study was likely to be similar to the population from which controls were selected. Second, similar to any other observational study, there is a potential for residual confounding. We minimized the confounding due to measurement error by using standardized methods for data collection and consistent definitions for variables in both patients with AMI and controls. We restricted patients with AMI to those with a first event to reduce the possibility that individuals with previous cardiovascular disease might have substantially changed risk factor levels before this event. Third, certain factors, such as diet, stress, illicit drug use, familial hyperlipidemia, history of congenital heart disease, pregnancy, and childbearing, were either not measured or were measured using inconsistent methods in VIRGO and NHANES; hence, we were unable to assess their associations with AMI in this study. As suggested by previous studies, these factors may be important contributors to AMI risk in young adults.^18,19^ Fourth, as with any other studies that use interviews to obtain medical history of participants, there may be recall bias in patients with AMI who tend to report family history more often than people without AMI. However, family history of AMI was measured by standardized questionnaire in both VIRGO and NHANES, and we used consistent definitions of risk factors in the 2 studies to minimize measurement error. Finally, the results of PAF need to be interpreted with caution, as patients with AMI in this study may have had different risk factor distributions compared with other patients with AMI.

## Conclusions

In this case-control study, 7 risk factors, many potentially modifiable, accounted for 85% of the risk of first AMI in young individuals. Significant differences in risk factor profiles and risk factor associations existed by sex and by subtypes of AMI. These findings suggest the need for sex-specific strategies in risk factor modification and prevention of AMI in young adults. Further research is needed to improve risk assessment of AMI subtypes.

## Notes

Supplement. **eMethods. **Methods to calculate population attributable fraction for individual risk factors **eTable 1.** AMI classification by the third global definition of MI **eTable 2. **Comparison of variables in VIRGO and NHANES
