# Supplementary material for: Sex-Specific Risk Factors Associated With First Acute Myocardial Infarction in Young Adults
Source: JAMA Netw Open. 2022 May 3;5(5):e229953. doi: 10.1001/jamanetworkopen.2022.9953 (PMC9066284; doi:10.1001/jamanetworkopen.2022.9953)
Supplement: Supplement. — eMethods. Methods to calculate population attributable fraction for individual risk factors eTable 1. AMI classification by the third global definition of MI eTable 2. Comparison of variables in VIRGO and NHANES [file jamanetwopen-e229953-s001.pdf]

Supplemental Online Content

Lu Y, Li SX, Liu Y, et al. Sex-specific risk factors associated with first acute myocardial infarction in young adults. *JAMA Netw Open*. 2022;5(5):e229953. doi:10.1001/jamanetworkopen.2022.9953

eMethods. Methods to calculate population attributable fraction for individual risk factors

eTable 1. AMI classification by the third global definition of MI

eTable 2. Comparison of variables in VIRGO and NHANES

This supplemental material has been provided by the authors to give readers additional information about their work.

© 2022 Lu Y et al. *JAMA Network Open.*

eMethods.

Methods to calculate population attributable fraction for individual risk factors.

Population attributable fraction (PAF) quantifies the fraction of AMI cases in the population that were attributable to an exposure to one or several risk factors.[1] In this study, provided the disease outcome AMI is relatively rare, PAF can be derived by substituting relative risk with a odds ratio estimated from a conditional logistic regression model. A clustered sandwich formula is used in the variance and confidence interval calculation.

PAF for a simple dichotomous exposure and disease, and no adjustment for confounding, was estimated using formula (1).

$$\boldsymbol{PAF_{unadj}} = P_d \times \frac{RR_{unadj}-1}{RR_{unadj}} \approx P_d \times \frac{OR_{unadj}-1}{OR_{unadj}} \qquad (1)$$

Where P_d is the proportion of cases exposed to risk factor, OR_{unadj} is the unadjusted odds ratio, estimated by the univarible conditional logistic regression model

PAF adjusted for confounding was calculated using formula (2).

$$\boldsymbol{PAF_{adj}} = P_d \times \frac{RR_{adj}-1}{RR_{adj}} \approx P_d \times \frac{OR_{adj}-1}{OR_{adj}} \qquad (2)$$

Where P_d is the proportion of cases exposed to risk factor, OR_{adj} is the adjusted odds ratio, estimated by the multivarible conditional logistic regression model

PAF for multiple risk factors was calculated using formula (3).

$$\boldsymbol{PAF_{multiple}} = 1 - \prod_{i=1}^{n}(1 - \mathrm{PAF_i}) \qquad (3)$$

Where n is the number of the multiple risk factors

© 2022 Lu Y et al. *JAMA Network Open.*

eReferences

1. Mansournia MA, Altman DG. Population attributable fraction. Bmj 2018;360.
2. Bruzzi, P., Green, S. B., Byar, D., Brinton, L. A., & Schairer, C. (1985). Estimating the population attributable risk for multiple risk factors using case-control data. American Journal of Epidemiology, 122(5), 904-914.
3. Rockhill B, Newman B, Weinberg C. Use and misuse of population attributable fractions. Am J Public Health. 1998 Jan;88(1):15-9. doi: 10.2105/ajph.88.1.15. Erratum in: Am J Public Health. 2008 Dec;98(12):2119. PMID: 9584027; PMCID: PMC1508384.

© 2022 Lu Y et al. *JAMA Network Open.*

eTable 1. AMI classification by the third global definition of MI.

Third Universal Definition of MI	Definition
Type 1	Plaque rupture, ulceration, fissuring, erosion, dissection with resulting thrombus
Type 2	Condition other than CAD contributes to imbalance between myocardial oxygen supply or demand
Type 3	Cardiac death with symptoms suggesting ischemia
Type 4a	Related to percutaneous coronary intervention
Type 4b	Stent thrombosis
Type 5	Related to coronary artery bypass graft
Unclassified	

AMI indicates acute myocardial infarction.

© 2022 Lu Y et al. *JAMA Network Open.*

eTable 2. Comparison of variables in VIRGO and NHANES.

**Risk factor**	**Ascertainment in VIRGO**	**Ascertainment in NHANES**	**Definition of variables used in analysis**
Marital status	In-person interview: Which best describes your current marital status?	In-person interview: Please describe your current marital status.	Marital status is classified as married/ living with partners vs. others (widowed, divorced, or separated; and never married).
Highest education level	In-person interview: What is the highest level of education you have completed?	In-person interview: What is the highest grade or level of school you have completed or the highest degree you have received?	Highest education level is classified as less than high school, high school, greater than high school.
Household income	In-person interview: What is your total household income (including those with whom you live, such as your spouse)?	In-person interview: Please describe your total household income (reported as a range value in dollars)	Annual household income is classified as <10,000, 10,000–99,999, and ≥100,000.
Health insurance	In-person interview: Do you have health care insurance?<br>[If Yes] What types of coverage do you have?	In-person interview: Are you covered by health insurance or some other kind of health care plan? [Include health insurance obtained through employment or purchased directly as well as government programs like Medicare and Medicaid that provide medical care or help pay medical bills.]	Individuals are classified as insured if they had any private health insurance, Medicare, Medicaid, military plan, government or state-sponsored health plan.
Hypertension	In-person interview:<br>• Has a doctor or other health professional ever told you that you have high blood pressure (excluding when you were pregnant)?<br>• Have you ever taken medicine for high blood pressure?<br>• Are you currently taking medicine for high blood pressure?	In-person interview:<br>• Have you ever been told by a doctor or other health professional that you had hypertension, also called high blood pressure?<br>• Because of your high blood pressure/hypertension, have you ever been told to take prescribed medicine?<br>• Are you currenly taking prescribed medicine for high blood pressure	Hypertension is defined based on self-reported previous diagnosis of hypertension or currently on antihypertensive medication.
Diabetes	In-person interview:<br>• Have you ever been told that you have diabetes (excluding when you were pregnant)?<br><br>Medical chart abstraction:<br>• Treatment of diabetes mellitus (check all that apply): None; diet; insulin; oral anti-diabetics<br>• HbA1c (%) in the most recent in past 3 months	In-person interview:<br>• Other than during pregnancy, have you ever been told by a doctor or health professional that you have diabetes or sugar diabetes?<br>• Are you now taking diabetic pills to lower your blood sugar? These are sometimes called oral agents or oral hypoglycemic agents.<br>• Are you now taking insulin?	Diabetes is defined based on previous physician diagnosis, or HbA1c ≥ 6.5%, or currently on antidiabetic medication.

© 2022 Lu Y et al. *JAMA Network Open.*

		Lab measurement: All participants ages 12 and older are given the option of a HbA1C% test during their physical examination	
Hypercholesterolemia	In-person interview:<br>• Has a doctor or other health professional ever told you that you have high cholesterol or abnormal levels of fats in your blood?<br>• Are you currently taking medicine for high cholesterol?<br><br>Medical chart abstraction:<br>• Lipids in the most recent in past 12 months	In-person interview:<br>• Have you ever been told by a doctor or other health professional that your blood cholesterol level was high?<br>• To lower your blood cholesterol, have you ever been told by a doctor or other health professional to take prescribed medicine?<br>• Are you now following this advice to take prescribed medicine?<br>Lab measurement: All participants ages 12 and older are given the option of a lipid test during their physical examination.	Hypercholesterolemia is defined based on previous physician diagnosis, or TC ≥200 mg/dL, or LDL-C ≥130 mg/dL, or HDL-C <40 mg/dL, or currently on lipid-lowering medication.
Obesity	Medical chart abstraction: Height and weight are measured during the index AMI admission for all patients.	Physical examination: Standing height is measured in participants ages 2 and older and weight is measured in participants at all ages.	We calculated body-mass index as weight in kilograms divided by the square of height in meters and obesity was defined as body mass index ≥ 30 kg/m^2.
Current smokers	In-person interview:<br>• Which of the following best describes your current cigarette smoking status?<br>• In the past 30 days, how many cigarettes did you smoke each day, on average?	In-person interview:<br>• On how many of the past 30 days did you/SP smoke a cigarette?<br>• During the past 30 days, on the days that you smoked, about how many cigarettes did you smoke per day?	Current smokers are defined as individuals who smoked any tobacco in the previous 30 days.
Regular alcohol intake	In-person interview:<br>• Do you ever drink beer, wine, liquor or any drink containing alcohol?<br>• On average how many days per week do you drink alcohol?<br>• On a typical day when you drink, how many drinks do you have?	In-person interview:<br>• In your} entire life, have you had at least 12 drinks of any type of alcoholic beverage?<br>• In the past 12 months, how often did you drink any type of alcoholic beverage? PROBE: How many days per week, per month, or per year did you drink?<br>• In the past 12 months, on those days that you drank alcoholic beverages, on the average, how many drinks did you have?	Regular alcohol intake is defined as alcohol consumption three or more times a week
Physical activity	In-person interview:<br>• Behavioral Risk Factor Surveillance Survey (BRFSS) physical activity instrument.<br>• Patients were asked if they participated in moderate or vigorous physical activity during the past 30 days. If they answered	In-person interview:<br>• Behavioral Risk Factor Surveillance Survey (BRFSS) physical activity instrument.<br>• Patients were asked if they participated in moderate or vigorous physical activity during the past 30 days. If they answered yes to either question, they were then asked the duration	Physical activity is classified into three levels: recommended (150 min or more of moderate physical activity or 75 min or more of vigorous physical activity per week), insufficient (between 10-149 min per week of moderate

© 2022 Lu Y et al. *JAMA Network Open.*

	yes to either question, they were then asked the duration and frequency of their participation in physical activity for an average week.	and frequency of their participation in physical activity for an average week.	physical activity or between 10-74 min per week of vigorous physical activity), or inactive (no participation or fewer than 10 min of moderate or vigorous physical activity per week).
History of congestive heart failure	Medical chart abstraction: history of congestive heart failure was extracted from medical record at the index AMI admission for all patients.	In-person interview:<br>Has a doctor or other health professional ever told you that you had congestive heart failure?	History of congestive heart failure is defined based on self-reported previous physician diagnosis of congestive heart failure
Family history of premature MI	In-person interview:<br>Have any of your blood relatives (including blood relatives, including grandparents, parents, brothers, and sisters) ever had a heart attack?<br>[If Yes] Which relatives and at what age did they first occur? [Complete all that apply.]	In-person interview:<br>Including living and deceased, were any of your close biological that is, blood relatives including grandparents, father, mother, sisters or brothers, ever told by a health professional that they had a heart attack or angina before the age of 50?	Family history of premature MI is defined as any of the participants' blood relatives ever had a heart attack before the age of 50.
Family history of diabetes	In-person interview:<br>Do any blood relatives in your immediate family have diabetes?	In-person interview:<br>Including living and deceased, were any of your biological relatives, that is, blood relatives, including grandparents, parents, brothers, and sisters, ever told by a health professional that they had diabetes?	Family history of diabetes is defined as any of the participants' blood relatives ever had diabetes.
Depression	In-person interview: Symptoms of depression that patients experienced in the past 2 weeks were assessed with the 9-item version of the Patient Health Questionnaire (PHQ-9)	In-person interview: Symptoms of depression that patients experienced in the past 2 weeks were assessed with the 9-item version of the Patient Health Questionnaire (PHQ-9)	Depressive symptom is defined as PHQ-9 score ≥ 10.
Menopause before age 45	In-person interview:<br>Have you reached menopause?<br>[If Yes] At what age?	In-person interview:<br>• What is the reason that you have not had a period in the past 12 months? (menopause is one of the options)<br>• What are your reasons for having used estrogen or progesterone? (menopause is one of the options)<br>• If menopause, at what age?	Early menopause is defined if a woman has reached menopause before age 45.

VIRGO indicates Variation in Recovery: Role of Gender on Outcomes of Young Acute Myocardial Infarction Patients Study; NHANES: National Health and Nutrition Examination Survey.

© 2022 Lu Y et al. *JAMA Network Open.*
